# Lateral Gene Expression in *Drosophila* Early Embryos Is Supported by Grainyhead-Mediated Activation and Tiers of Dorsally-Localized Repression

**DOI:** 10.1371/journal.pone.0029172

**Published:** 2011-12-22

**Authors:** Mayra Garcia, Angelike Stathopoulos

**Affiliations:** Division of Biology, California Institute of Technology, Pasadena, California, United States of America; Stockholm University, Sweden

## Abstract

The general consensus in the field is that limiting amounts of the transcription factor Dorsal establish dorsal boundaries of genes expressed along the dorsal-ventral (DV) axis of early *Drosophila* embryos, while repressors establish ventral boundaries. Yet recent studies have provided evidence that repressors act to specify the dorsal boundary of *intermediate neuroblasts defective* (*ind*), a gene expressed in a stripe along the DV axis in lateral regions of the embryo. Here we show that a short 12 base pair sequence (“the A-box”) present twice within the *ind* CRM is both necessary and sufficient to support transcriptional repression in dorsal regions of embryos. To identify binding factors, we conducted affinity chromatography using the A-box element and found a number of DNA-binding proteins and chromatin-associated factors using mass spectroscopy. Only Grainyhead (Grh), a CP2 transcription factor with a unique DNA-binding domain, was found to bind the A-box sequence. Our results suggest that Grh acts as an activator to support expression of *ind*, which was surprising as we identified this factor using an element that mediates dorsally-localized repression. Grh and Dorsal both contribute to *ind* transcriptional activation. However, another recent study found that the repressor Capicua (Cic) also binds to the A-box sequence. While Cic was not identified through our A-box affinity chromatography, utilization of the same site, the A-box, by both factors Grh (activator) and Cic (repressor) may also support a “switch-like” response that helps to sharpen the *ind* dorsal boundary. Furthermore, our results also demonstrate that TGF-β signaling acts to refine *ind* CRM expression in an A-box independent manner in dorsal-most regions, suggesting that tiers of repression act in dorsal regions of the embryo.

## Introduction

During development the embryo is patterned by the localized expression of genes to discrete parts of the embryo. Such tight spatial regulation of gene expression is necessary to set the boundaries that distinguish different cell types required for proper development. One mechanism to impart spatial information is to regulate gene expression through transcription factors that are spatially localized. Alternately, localized activation of signaling pathways in particular domains can also influence the boundaries of gene expression.

In *Drosophila melanogaster*, the dorsal-ventral (DV) axis of the pre-gastrula embryo is patterned by a nuclear gradient of the NF-κB homologous transcription factor Dorsal [1]. High levels of nuclear Dorsal are present in ventral regions of the *Drosophila* embryo and nuclear levels decrease progressively toward more dorsal regions. Due in part to these differing nuclear Dorsal levels, different domains of gene expression are established along the DV axis to specify different cell types [2]. In the ventral most regions of the embryo, high concentrations of nuclear Dorsal drive expression of genes such as *twist* and *snail (sna)* to specify the presumptive mesoderm. In ventral lateral regions of the embryo, intermediate levels of Dorsal activate genes such as *rhomboid (rho)* and *ventral neuroblast defective* (*vnd*) and low levels of Dorsal support expression of genes such as *short gastrulation (sog)* in broad lateral domains of the embryo (that encompass both ventral-lateral and dorsal-lateral regions) to specify distinct domains within the presumptive neurogenic ectoderm [3], [4], [5]. Lastly, as Dorsal can also function as a repressor, the expression of some genes such as *zerknüllt* (*zen*) are limited to dorsal regions of the embryo, leading cells in this domain to adopt amnioserosa and non-neurogenic dorsal ectoderm cell fates [2], [6], [7]. Even though Dorsal provides positional information through its dorsal-ventrally modulated nuclear gradient, combinatorial interactions of transcription factors are very influential towards DV patterning. Specifically, Dorsal regulates gene expression together with other transcription factors, such as the bHLH factor Twist and the early ubiquitous activator Zelda [8], [9], [10].

More and more evidence suggests that signaling pathways also help to define gene expression patterns in the early embryo. For example, the expression domains of several Dorsal target genes cannot be explained by changing Dorsal levels (and/or the localization of any other previously characterized transcription factors). Additionally, it is well understood that signaling molecules provide positional information to help define the very specific expression domain encompassed by the gene *single-minded (sim)*. *sim* is expressed as a stripe of a single cell width present in ventrolateral regions of the embryo, within cells located between the presumptive mesoderm and neurogenic ectoderm boundary. *sim* expression is supported by combinatorial interactions of Dorsal and Twist transcription factors and also through Notch-dependent signaling [11].

Along similar lines, the gene *intermediate neuroblast defective (ind)* is expressed in dorsal-lateral regions of the embryo in a stripe of 5–7 cells in width, which is narrower than the broad domain encompassed by *sog*. Genetic studies support the view that refined *ind* expression is supported by inputs from both Dorsal and Epidermal growth factor receptor (Egfr) signaling, suggesting that limiting amounts of both of these inputs help delineate *ind* expression boundaries [12]. The Egfr gene is ubiquitously expressed in embryos but the receptor is activated locally in ventrolateral regions by the ligands Vein and Spitz [13], [14]. Several binding sites for the ETS transcription factor, which mediates Egfr signaling, are also found in the *ind* cis-regulatory module CRM, but it has not been shown if they are required for activation or whether an indirect mechanism is used for activation of *ind* expression via Egfr signaling [15].

No other gene in the *Drosophila* embryo described to date shares the same expression domain as *ind*, yet understanding how the *ind* expression domain is regulated may have far-reaching implications. Interestingly, the genes that pattern the ventral nerve cord of *Drosophila* and the neural tube of higher vertebrates share a conserved organization and function [16], [17]. Specifically, the gene *ventral neuroblast defective* (*vnd*)/*Nkx2.2* is expressed ventral to *ind/Gsh*, and the gene *muscle specific homeobox* (*msh*)/*Msx1/2* is expressed dorsally to *ind* [18], [19], [20]. Experiments conducted in the *Drosophila* embryo have suggested that the ventral boundaries of these genes are set following a “ventral dominance rule”, in which the more ventral genes repress expression of the more dorsal genes [21]. In contrast, it had been proposed that the dorsal boundaries of these genes result from limiting amounts of the activator, Dorsal, present in distinct domains along the DV axis [2]. However, recently it was discovered that the *ind* gene is expressed in a domain along the DV axis where the Dorsal gradient appears uniform without a clear transition that would be capable of setting a dorsal border [22]. A previous analysis of the *ind* CRM suggested evidence for a dorsally-acting repressor which could explain how the dorsal boundary of *ind* is specified [15].

Direct evidence for repressor action within dorsal regions of the early embryo was found through analysis of the *cis*-regulatory region of *ind* [15]. A 1.4 kB DNA fragment located ∼2 kB downstream of the *ind* coding sequence was found to support expression in a refined stripe within lateral regions of the embryo, in a pattern comparable to the endogenous gene. However, the promoter proximal half of the *ind* CRM drove expression of a reporter gene within a broad pattern, one that extends into ventral-lateral as well as dorsal-lateral regions, suggesting that the distal half contains repressor binding sites. Using a chimeric CRM assay designed to detect repression along the dorsal-ventral axis by silencing of an associated *even-skipped stripe 3/7* CRM (*eve.stripe3/7*), this previous study found that the 1.4 kB *ind* CRM mediates repression of *eve.stripe3/7* in dorsal and ventral regions of the embryo. A specific search for an element supporting dorsal repression was conducted and identified a 111 base pair (bp) region of the *ind* CRM, which supported dorsal-lateral and dorsal repression of *eve.stripe3/7*. A 12 bp sequence was highlighted, as it repeats twice within these 111 bp, and was called the A-box (WTTCATTCATRA). Importantly, in this previous study, when the A-box was mutated in the context of a minimal element supporting repression in dorsal regions (i.e. 267 bp fragment), repression of the *eve.stripe3/7* CRM was lost. Presumably transcription factors bind to the A-box element to help establish the dorsal boundary of the *ind* gene, but their identities remained unknown.

Additional evidence also suggests that TGF-ß signaling may also regulate the *ind* expression domains, but whether or not this signaling pathway functions through the A-box element was not known. Decapentaplegic (Dpp) is a TGFß/BMP homolog that is limited in its expression to dorsal regions of the embryo and functions as a morphogen to support patterning of the amnioserosa, at higher levels in dorsal-most regions of the embryo, and the non-neurogenic ectoderm, at lower levels in dorsal-lateral regions of the embryo [23]. A previous study found that in mutants in which Dpp signaling is expanded into lateral regions of the embryo, *ind* expression is lost [12]. Likewise, ectopic expression of *dpp* in lateralized embryos that exhibit expanded *ind* expression throughout the embryo was able to repress *ind* in the domain where Dpp signaling was presented [17]. Also, the *ind* CRM contains a 15 bp DNA sequence implicated in TGF-β signaling-mediated repression [15]. Similar sites have been shown to mediate repression by recruiting a Dpp-dependent Schnurri/Mad/Medea (SMM) protein complex, but SMM dependent repression of *ind* has never been shown and in fact this mechanism of repression has only been shown to act at later stages of development [24], [25].

Therefore, to gain further insight into how patterning is controlled along the dorsal-ventral axis of *Drosophila* embryos, we tracked the repression activity supported by different DNA elements associated with the *ind* CRM. We found that the A-box element facilitates both activation and repression of *ind* and propose that this helps to mediate a sharp border. In addition, we found that TGF-β signaling supports *ind* repression in dorsal-most regions of the embryo through the SMM site located within the *ind* CRM that is distinct from the A-box.

## Results

### Chimeric CRM assays can help identify and track repression activity associated with CRM sequences

**Figure 1:**
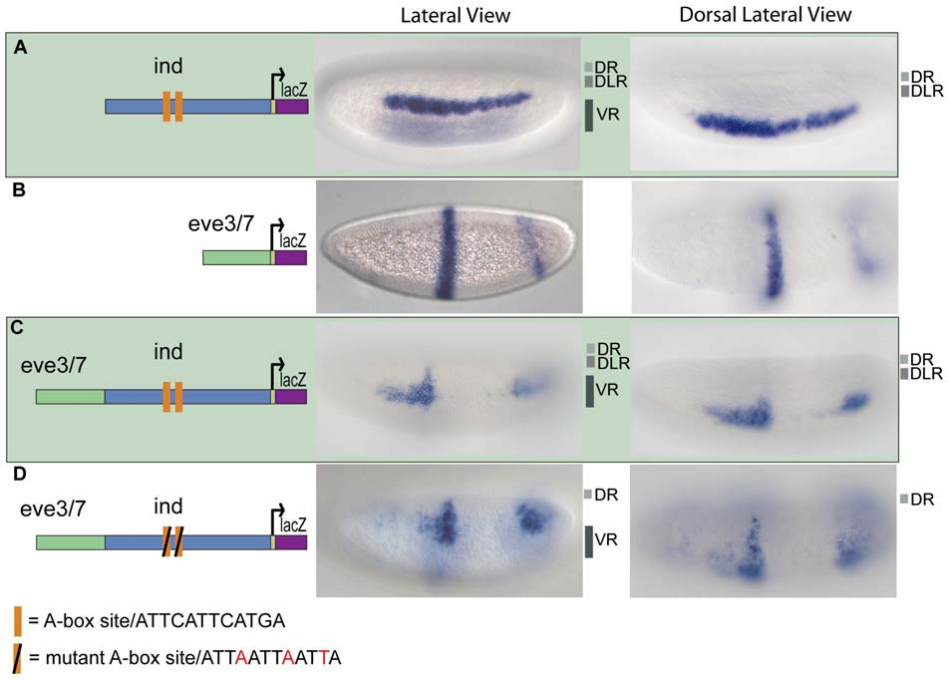
The *ind* CRM contains binding sites that mediate repression in dorsal regions. *lacZ* reporter expression was visualized within cellularized embryos (late stage 5) by in situ hybridization using a digoxigenin-labeled antisense *lacZ* riboprobe. In this and all subsequent figures, embryos are oriented with anterior to the left. In addition, embryos are oriented to show views of lateral, dorsal on top, (left image) and dorsal (right image) domains. The repression domains are outlined to the right of each image: DR = dorsal repression, DLR = dorsal lateral repression, and VR = ventral repression. The schematic depicts the chimeric CRM combinations used: (A) 1.4-kb *ind* CRM drives expression of *lacZ* a 5–7 cell lateral stripe representative of *ind* expression; (B) 0.5 kb *eve.stripe3/7* CRM drives expression of *lacZ* in two anterior-posterior stripes representative of *eve.stripe3/7* expression; (C) *eve.stripe3/7-ind* chimeric CRM drives expression of *lacZ* in a non-additive fashion showing repression of *eve.stripe3/7* in dorsal, dorsal lateral, and ventral regions; (D) *eve.stripe3/7-mut-A-box-ind* chimeric CRM supports non-additive expression with repression of *eve.stripe3/7* in dorsal and ventral regions but not dorsal lateral regions.

In order to gain insights into how the boundaries of dorsal-ventral patterning genes are set, we deconstructed the *cis*-regulatory element of *ind* to find direct evidence for dorsal repression activity. We utilized a chimeric cis-regulatory module (CRM) assay, using *eve.stripe3/7* and *ind* CRMs in order to determine whether repressors are present within either of these sequences to help refine the domains of expression [15]. The *ind* CRM supports expression along the DV axis in a lateral stripe, comparable to the endogenous gene (Figure 1A) [15]. In turn, the *eve.stripe3/7* sequences supports expression of two stripes located along the anterior-posterior (AP) axis of embryos (Figure 1B) [26]. When two CRMs are placed in tandem upstream of a reporter gene (i.e. *lacZ*), if additive expression is observed this result indicates that either repressors are not present or they are not located in range to act on the adjacent CRM; conversely, if non-additive expression is observed this indicates repressors are present and function to silence activators associated with both CRMs. Previously, using a chimeric CRM assay, it was shown that the 1.4-kB *ind* CRM drives repression of *eve.stripe3/7* (Figure 1C) [15]. In this case non-additive expression is observed; the *eve.stripe3/7* CRM is repressed in ventral regions by *snail* and *vnd* repressor sites located in the *ind* CRM and by unknown transcription repressors in dorsal regions. Concurrently, the *ind* CRM is repressed by Knirps, through sites in the *eve.stripe3/7* CRM, forming a gap in the *ind* expression pattern. It was suggested the unknown transcription repressors located in dorsal regions act through a pair of 12-bp A-box sequences located within the 1.4-kb *ind* CRM. Here we examined the function of the A-box sequence more closely.

### The A-box element mediates repression of *ind* in dorsal-lateral regions of the embryo, while other sequences support repression in ventral and dorsal-most regions of the embryo

**Figure 2:**
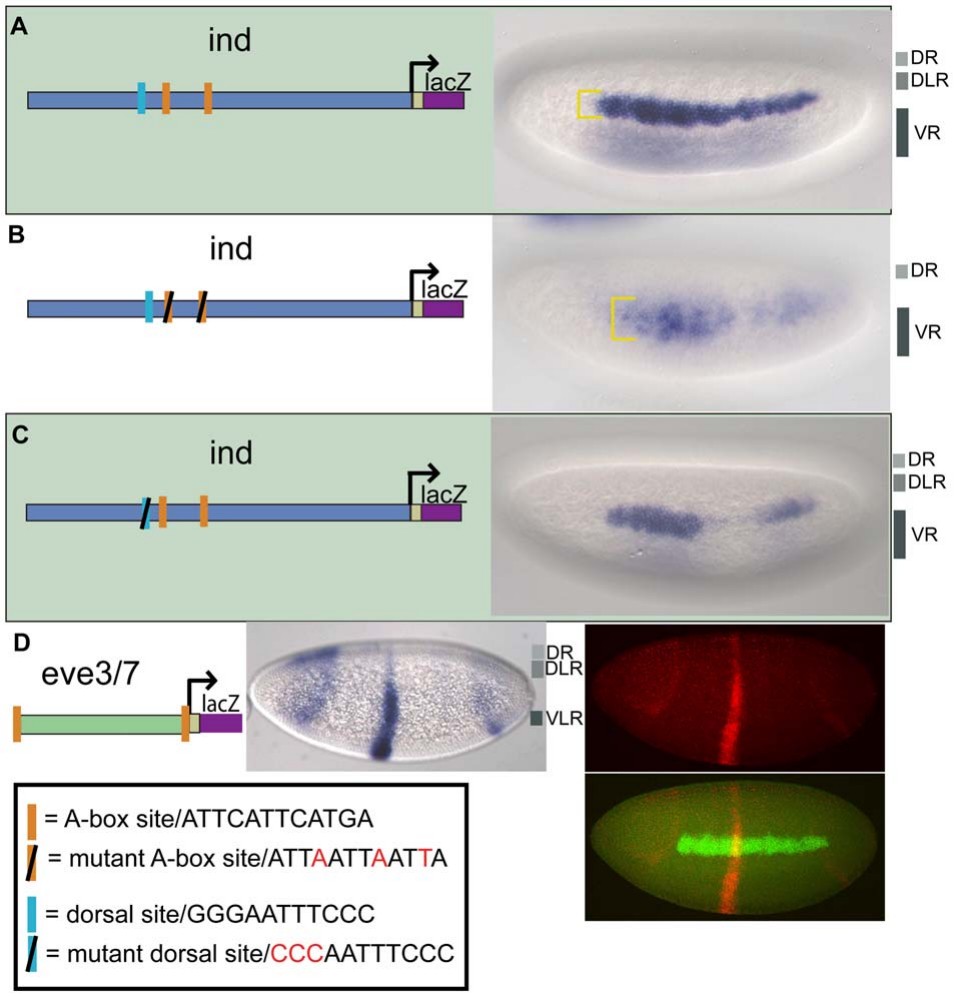
The A-box sites are necessary for dorsal lateral repression and sufficient for dorsal and dorsal-lateral repression. Site-directed mutagenesis was used to mutate regulatory sites in the *ind* CRM. The CRMs depicted in the schematic were used to drive expression of *lacZ* in embryos that were analyzed by in situ hybridization using a *lacZ* anti-sense riboprobe. Cellularized embryos of stage 5 are oriented to show a lateral view, with anterior to the left and dorsal on the top. The yellow brackets mark the height of the expression pattern. The repression domains are outlined to the right of the image: DR = dorsal repression, DLR = dorsal lateral repression, VR = ventral repression, VLR = ventral lateral repression. (A) 1.4 kB *ind* CRM drives expression of a lateral stripe of 5–7 cells in width comparable to *ind* expression. (B) 1.4 kB *mut-A-box-ind* CRM drives expression of 7–10 cell width lateral stripe that is diffuse, weak, and expanded compared to the *ind* CRM. (C) 1.4 kB *mut-dorsal-ind* CRM drives expression that has a gap and is weak in posterior regions compared to the *ind* CRM. (D) *eve.stripe3/*7 CRM flanked by A-box sites (*A-box-eve.stripe3/7-A-box*) shows repression in dorsal, dorsal-lateral, and ventral-lateral regions. In the fluorescent image. *lacZ* expression is shown in red and endogenous *ind* expression is shown in green as detected by multiplex fluorescent in situ hybridization [58].

When we mutated both of the A-box sites in the context of the full-length *ind* CRM and assayed the fragment's ability to repress expression of the associated *eve.stripe3/7* CRM, repression of *eve.stripe3/7* was lost in dorsal lateral regions (Figure 1D, compare with 1C). This result demonstrated that these two A-box sequences are necessary to mediate dorsal-lateral repression of *eve.stripe3/7* by the *ind* CRM. Next, we assayed the full-length *ind* CRM with two mutant A-boxes alone and found that *lacZ* reporter expression was expanded into dorsal-lateral regions; giving a broad, patchy, and diffuse pattern not a sharp stripe of 5–7 cells in width representative of *ind* (Figure 2B, compare with 2A).

However, even in the absence of the A-box sites, repression was retained in dorsal-most and ventral regions of the embryo when the A-box was mutated in the context of the full-length CRM (Figure 2B), as well as in the chimeric CRM assay of *ind* and *eve.stripe3/7* CRMs (Figure 1D). These results suggest that the A-box sequences mediate dorsal-lateral repression, but that there might be other repressor binding sites in the *ind* CRM which mediate repression in dorsal-most and ventral regions of the embryo. Vnd and Snail binding sites within the *ind* CRM most likely mediate the repression observed in ventral regions [21]. In contrast, while we were able to track repression in dorsal-most regions, the identity of the responsible transcription factors was unknown.

### A-box elements limit expression in dorsal-lateral and dorsal regions of embryos

Another important question is whether the A-box elements are sufficient to cause repression of the *eve.stripe3/7* CRM, as perhaps multiple sequences within the *ind* CRM are necessary to support repression. To investigate this, we flanked the *eve.stripe3/7* CRM with the A-box element (i.e. A-box.*eve.stripe3/7*.A-box) and observed clear repression in dorsal-lateral regions, as expected, and also within dorsal regions of the embryo (Figure 2D). Weak repression was also observed in ventrolateral regions at lower frequency (data not shown). This result suggests that A-box sequences are sufficient to support repression in dorsal-lateral regions, but also contribute to repression in dorsal-most and ventrolateral regions of the embryo.

The expression supported within the *eve.stripe3/7* domain did extend a few cells above the endogenous *ind* dorsal boundary in the context of the A-box.*eve.stripe3/7*.A-box reporter. This may indicate the chimeric CRM assay is limited in its ability to track repression activity as the stripe of expression also extended a few cells above *ind* when the full length *ind* CRM was assayed in tandem to *eve.stripe3/7*. Alternatively, sharp definition of the *ind* dorsal boundary may require more input than localized repressor activity.

### The Dorsal transcription factor only partially supports activation of *ind*

We investigated the activation of the *ind* expression pattern by mutagenizing the sole match to the Dorsal binding site consensus present within the *ind* 1.4 kB CRM (Figure 2C). *ind* is not expressed in *dorsal* mutants [12], thus, we expected loss of the sole Dorsal binding site would severely impair reporter expression. Instead, we found that the expression pattern driven by the mutated CRM is very similar to that driven by the wild-type CRM, except for a gap in the expression pattern (Figure 2C).

Early *ind* expression, at the start of cellularization, exhibits a smaller gap in expression at 40% egg length [15] which is likely mediated by anterior-posterior patterning factors. In reporter constructs, repression within this domain is more apparent with the 1.4 kb *ind* CRM sequence is oriented in the opposite direction relative to the promoter in reporter constructs (data not shown). The function of activators, including Dorsal and others that act through the A-box sequence, are likely required to counterbalance this repression.

Our results suggest that Dorsal binding contributes to *ind* activation but that other activators also influence *ind* expression. Furthermore, chromatin immunoprecipitation (i.e. ChIP-seq) experiments did not detect Dorsal binding in the genome at the *ind* CRM [27], which indicates Dorsal may not bind to the *ind* CRM (or that it is a very transient interaction). Collectively, these results suggest that additional transcriptional activators likely function to support *ind* expression.

### Dorsalized and lateralized embryos provide insights into the localization of the A-box repressor activity

Next we introduced the *lacZ* reporter gene containing the *eve.stripe3/*7 CRM flanked by A-box sequences (i.e. A-*box-eve.stripe3/7-A-box*) into different mutant backgrounds to test whether the repressor activity associated with the A-box sequence is influenced by altered DV positional information. Maternal mutant backgrounds exist that affect the levels of nuclear Dorsal (i.e. low or absent) to create lateralized or dorsalized embryos, respectively. Expression of Dorsal target genes are affected such that certain genes expressed by a particular level of Dorsal, normally refined in expression to distinct domains along the DV axis, are instead expressed ubiquitously or absent in either of these mutant backgrounds. In sum, our aim was to determine whether the repressor activity was responsive to changes in Dorsal levels, providing additional evidence that the repressor activity we had tracked was indeed functioning in a DV localized manner.

**Figure 3:**
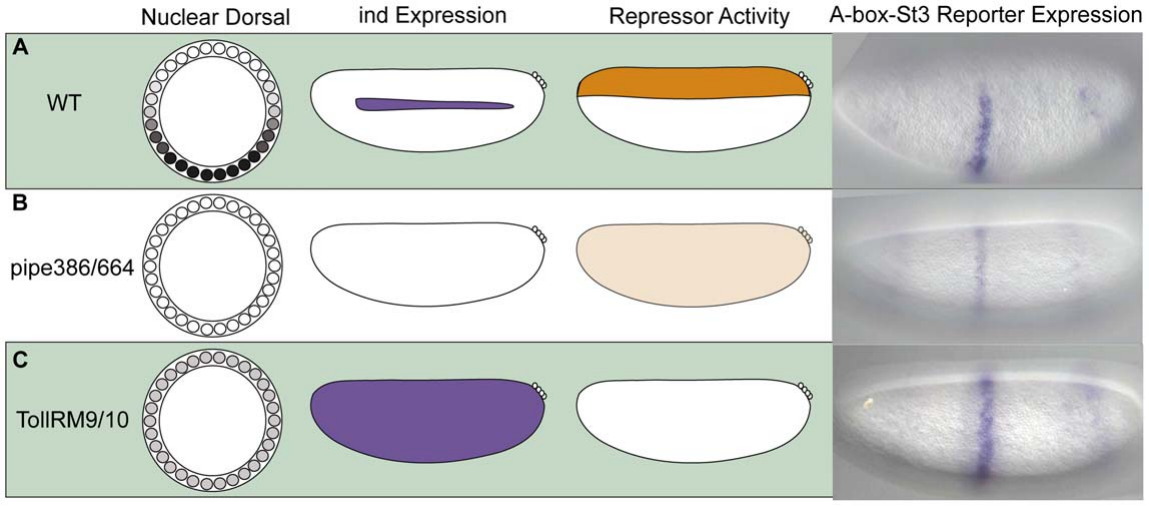
Dorsalized and lateralized embryos provide insights into the A-box repressor domain of activity. The depictions show the Dorsal nuclear gradient within embryo cross-section schematics, whereas *ind* expression and the putative repressor activity are schematized within lateral views. Expression of the *A-box-eve.stripe3/7-A-box* reporter gene was examined by in situ hybridization in (A) wild-type, (B) *pipe384/pipe664* mutants, and (C) Toll^RM9^/Toll^RM10^ mutants. The in situ images show *lacZ* expression as such: (A) Repression of *lacZ* is shown in dorsal regions of the embryo in WT embryos. (B) Weak repression of *lacZ* is shown throughout the embryos from *pipe* mutant females (i.e., dorsalized embryos). (C) A lack of repression of *lacZ* is shown in embryos from Toll^RM9/10^ mutant females (i.e., lateralized embryos).

In *pipe* mutants, Dorsal is not able to enter the nucleus thus Dorsal target genes are not activated, resulting in dorsalized embryos [2], [28]. In this mutant background, endogenous *ind* is not expressed. We assayed the *A-box-evestripe3/7-A-box lacZ* reporter construct in the *pipe* mutant background and found that expression of *lacZ* was retained but severely dampened (Figure 3B compare with 3A). This result suggests that some repressor activity is present ubiquitously in dorsalized embryos but most likely it is less active, because only partial repression of the reporter is observed.

We also examined reporter expression in *Toll^rm9/10^* embryos, which have a partially active form of the Toll receptor allowing low levels of Dorsal to enter the nucleus throughout the embryo [2]. In this background *ind* is expressed throughout the embryo, suggesting that repressors are unable to refine the *ind* pattern in this background. We also observed strong uniform expression of the *lacZ* reporter in the *eve.stripe3/7* domain indicating that in this background the repressor activity is gone (Figure 3C).

The A-box element clearly supports repression in dorsal regions of the embryo and is responsive to mutations altering DV pattern (Figure 3). These results suggest the A-box associated repressor exhibits localized expression in dorsal regions of the embryo and/or that its activity is modulated by signaling pathways that exhibit differential activation along the DV axis.

### Affinity chromatography and mass spectrometry identifies putative A-box binding factors

In order to provide molecular insight into the mechanism by which *ind* expression in dorsal regions is limited, we set-out to identify the factor that binds the A-box element choosing affinity chromatography using a 22 bp oligonucleotide containing the A-box sequence (12 bp) and endogenous flanking regions (5 bp on either side). As a control, we also compared binding with that obtained with a mutant A-box sequence modified in 3 of 12 bp, which we showed does not support dorsal repression when assayed in the context of a chimeric CRM assay *in vivo* (see Figures 1D, 2B) and containing different flanking region].

**Figure 4:**
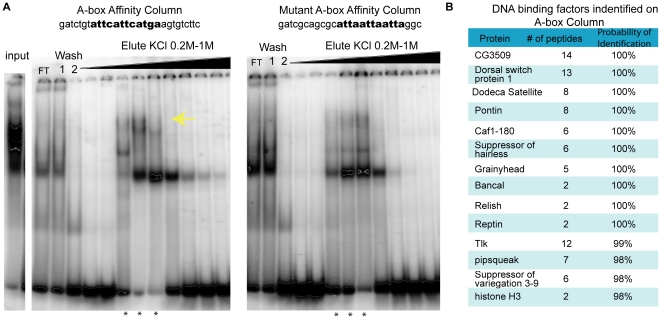
Affinity chromatography and mass spectrometry was used to identify factors that bind the A-box element. (A) shows the EMSAs preformed using γ^32^P-labeled A-box oligonucleotides on nuclear extract fractions after they were affinity purified with the A-box column and the mutant A-box column. FT denotes the flow through which did not bind to the column. The black arrow marks the area where the A-box specific binding was found. The stars mark the samples used for mass spectrometry identification. (B) The table lists the DNA binding factors that bound to the A-box element column but not the mutant A-box column. The “# of peptides” corresponds to the number of unique peptides that contributed to the protein identification. The probability of identification was calculated by the program Scaffold used to identify the proteins by mass spectrometry analysis and corresponded to the likelihood a correct match was made.

We used affinity chromatography to purify proteins that recognize the A-box or mutant A-box sequence from early embryonic nuclear extracts age 0–6 hours. The A-box binding activity was tracked throughout a number of biochemical separations (see Figure S1 and materials and methods). There were several factors that bound to both columns but some of the binding was specific to the A-box (Figure 4A). Cold competition with the A-box versus the mutant A-box confirmed the binding observed was specific to the A-box (data not shown). With advances in mass spectroscopy, we could analyze a complex sample containing a number of proteins. Therefore, at this step, we analyzed samples isolated from either the A-box column or the mutant A-box column by mass spectrometry.

Focusing on factors that only bound the A-box column (Figure 4B), we selected targets for future analysis. Several transcription factors were found specifically associated with the A-box, and not the mutant A-box column. Furthermore, several chromatin-related factors bound to the A-box column but failed to bind the mutant A-box column (Figure S2). This suggested to us that the repressor activity associated with the intact A-box sequence may be comprised of a large complex of proteins including chromatin components; a role for chromatin in supporting expression in the early *Drosophila* embryo is unclear (see Discussion).

### The Grainyhead transcription factor binds to the A-box sequence and is required to support *ind* expression

In order to narrow down a list of factors to examine in this preliminary analysis, we focused on identifying factors that bind specifically to the A-box DNA sequence. We conducted EMSAs on the following factors, which contain a predicted DNA-binding domain, and for which cDNAs were available: ATP-dependent chromatin assembly factor large subunit (Acf1), Structure specific recognition protein (Ssrp), CG3509, Grainyhead (Grh), Dorsal switch protein 1 (Dsp1) and Pipsqueak (Psq) (data not shown). Of these factors, only Grh exhibited binding to the 22 bp oligonucleotide, containing the 12 bp A-box and endogenous sequences.

**Figure 5:**
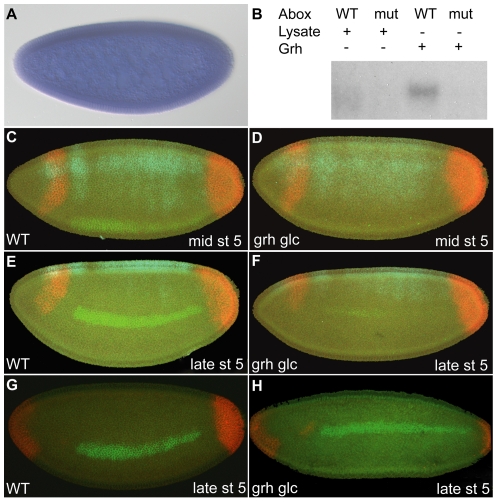
Grainyhead binds the A-box element and is involved in activation of *ind*. (A) *Grh* is expressed ubiquitously in embryos as detected by in situ hybridization using a *grh* riboprobe. (B) Grh was expressed in rabbit reticulocytes and EMSA was performed using γ^32^P-labeled A-box and mut-A-box oligonucleotides. Grh bound the A-box oligo but did not bind the mutant A-box oligo. Reticulocyte lysate alone was also tested for binding as a control. Expression of *zen* (cyan), *tll* or *hkb(G and H)* (red) and *ind* (green) are shown in wildtype (C, E, and G) and *grh* glc derived embryos (D, F, and H). The embryos in C and D are tilted ventrally to show the broad *zen* expression indicative of mid stage 5. Weak *ind* expression is observed in WT embryos (C) but not in embryos derived from *grh glc* (E). The embryos in E, F, G and H are oriented to show a lateral view and are late stage 5. Strong *ind* expression was detected in wildtype (WT) embryos (E and G) while very faint (F) or thin (H) *ind* expression was detected in embryos derived from *grh* glc females.

Using in vitro translated proteins in EMSAs, we further analyzed Grh and found that while it bound the A-box element it did not bind to the mutant A-box element (Figure 5B, full gel Figure S3). We, therefore, conducted additional analysis on Grh as it seemed a likely candidate to support the A-box repression activity. The *grh* gene is maternally and zygotically expressed [29], [30], and by in situ hybridization we confirmed that it is ubiquitously expressed in the early embryo (Figure 5A). While some evidence exists that *grh* transcripts are localized to dorsal and lateral regions of the embryo (Huang, 1995), we could not detect such a localized expression domain by in situ hybridization even though a number of different riboprobes were designed to detect *grh* transcripts.

We generated *grh* germline clone females in order to deplete both maternal and zygotic *grh* expression from embryos. The conventional method of creating germline clones [31], which relies on flipase catalyzed mitotic recombination in the context of transheterozygous FRT *ovoD* (dominant female sterile mutation) and FRT *grh* chromosomes, for example, could not be used because *ovoD* within the commonly used FRT *ovoD* chromosome is most likely inserted at the *grh* locus. FRT *ovoD* in combination with all *grh* alleles tested are zygotically lethal, but no lethality was observed with *ovoD* insertions located on other chromosomes. Thus, it was necessary to make germline clones in females of the genetic background FRT *grh*/FRT GFP. Embryos obtained from these females were manually screened for absence of GFP [32], thus allowing isolation of embryos containing the mutant form of *grh*. To ensure that *grh* zygotic transcripts were absent, females containing germline clones were mated to males containing appropriate balancer chromosomes to allow detection in the early embryo (i.e. FRT *grh*/Cyo *ftz-lacZ*; see Materials and Methods).

Because manual hand sorting of embryos was required, only a small number of embryos could be examined, but multiplex in situ hybridization allowed us to examine the expression of multiple genes simultaneously. Therefore, in addition to examining the effect of loss of *grh* on *ind* expression, we also assayed whether this mutation affected expression of two other genes, *tailless* (*tll*) and *zen*. In a previous study, embryos devoid of *grh* maternal message were produced X-ray irradiation induced mitotic recombination; *tll* was found to be expanded in *grh* mutant embryos obtained in this manner [33]. However, we failed to see expansion of *tll* in embryos lacking both maternal and/or zygotic *grh*; a similar negative result was recently reported [34]. Our results concur with those of Harrison et al. and we agree that the expansion of *tll* observed previously (Liaw et al., 1995) was most likely an artifact induced by X-ray irradiation. We also examined *zen* expression in order to determine if there was any effect on Dpp target genes due to loss of *grh*; a previous study had suggested that *grh* may be involved in repression of *dpp* [29]. During early stages, *zen* expression is broad, present in dorsal-lateral regions as well as dorsal regions, but by cellularization (late stage 5) its pattern has refined to a dorsal stripe present in dorsal-most regions of the embryo [35]. This later pattern is regulated by Dpp-mediated TGF-β signaling [36], [37]. However, no effect on *zen* expression was identified in embryos lacking maternal and zygotic *grh* (Figure 5F, compare 5D; and data not shown).

In contrast to the “normal” expression patterns of the genes *tll* and *zen* within *grh* mutant embryos, we found that *ind* expression was severely dampened in these mutants (Figures 5C, E, G compare with 5D, F, H); the data for grh^IM^ is shown. In wild-type embryos, *ind* comes on weakly at first during early stage 5 (precellularization), but by the end of stage 5 upon complete cellularization of embryos *ind* expression becomes sharp and clearly apparent. In the absence of maternal *grh*, the *ind* pattern was severely to weakly affected (Fig. 5 compare F to H), with some embryos showing an almost complete loss of *ind* in late stage 5 and others showing a weak thin uniform stripe compared to the wild-type tapered stripe. It is possible that the *grh* zygotic contribution relates to the variability. Furthermore, only a weak phenotype was observed with the grh^B37^ allele, which is expected because grh^IM^ is the stronger amorphic allele. To confirm that the phenotype observed was due to the grh^IM^ mutation and not a secondary mutation, we mated the females containing the germline clones to males in which the *grh* gene is absent, *Df(2R)Pcl7B/Cyoftzlacz*. We did not observe a rescue suggesting the phenotype is associated with loss of *grh*.

**Figure 6:**
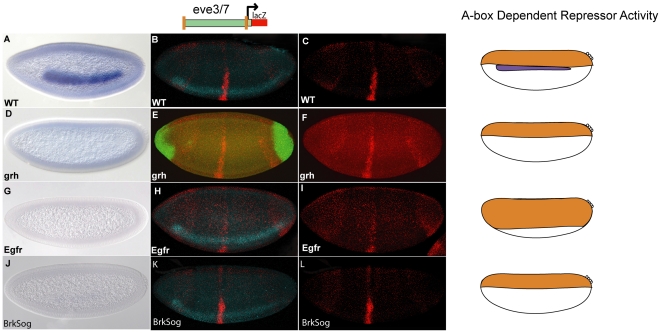
Analysis of A-box dependent and A-box independent repression in different mutant backgrounds. Embryos (stage 5) were analyzed by in situ hybridiation for *ind* expression. Multiplex in situ hybridization was used to analyze A-box dependent and A-box independent repression in different mutant backgrounds. The schematic shows the CRMs used to drive expression of *lacZ*. The orange boxes in the schematic correspond to A-box sites while the orange boxes with a slash through them correspond to mutant A-box sites. The cartoons to the right of the images show where A-box/Cic dependent (orange) and A-box independent (green) repression are located in WT embryos and in the corresponding mutants; *ind* is only expressed in wildtype (purple). *ind* expression is shown in WT embryos (A), *grh* glc derived embryos (D), *egfr* mutants (G), and *brk sog* double mutants (J). The *A-box-eve.stripe3/7-A-box* reporter construct was introduced into different mutant backgrounds and analyzed by in situ hybridization; *lacZ* (red), and *vnd* (blue) is shown in a in WT embryo (B), *grh* glc derived embryo shows expression of *hkb* (green) rather than *vnd* and is tilted dorsally relative to the rest of the embryos (E), *egfr* mutant (H) and *brk sog* mutant (K). For clarity *lacZ* expression is shown alone for the corresponding embryos WT (C), *grh* glc (F), *egfr* mutant (I), and *brk sog* mutant (L). The same microscope settings were used to image C, I, and L; different settings were used for F but it was compared to a WT embryo taken under the same settings (not shown).

To investigate whether Grh is responsible for the repressive function as well as the activation function of the A-box, we assayed whether the A-box could support repression in embryos obtained from *grh* mutant germline clone females. We did not see an effect on the repressor activity supported by an *eve.stripe3/7* CRM flanked by A-box sites in the absence of maternal and zygotic *grh*; the pattern was repressed in dorsal regions even in the absence of *grh* (Figure 6 E and F).

The loss of *ind* expression in *grh* mutants and retention of dorsally-localized repression was unexpected because we had isolated the Grh protein using the A-box element, which clearly supports repression in dorsal regions of the embryo. Nevertheless, we had observed that mutagenesis of the A-box sites within the *ind* CRM not only caused expansion of the pattern but also caused a reduction in levels of expression of the reporter gene (Figure 2B, compare with 2A). Therefore, we reasoned that Grh might function as a transcriptional activator that drives *ind* expression through the A-box sequence, and hypothesized that yet another factor might bind to the same site, to mediate repression. A recent study shed light on this issue as it presented evidence that the Capicua (Cic) repressor is required to support repression through the A-box and that it is modulated by Egfr signaling [38].

### Loss of Egfr signaling expands the A-box supported repression domain ventrally

To gain insights into the mechanism of repression, we examined *ind* expression as well as A-box mediated repression in *cic* as well as *Egfr* mutants. First we looked at *Egfr* mutants in which it has been shown that *ind* expression is lost (Figure 6E) [12]. Egfr signaling supports *ind* expression either directly by supporting activation through the various ETS sites found in the *ind* CRM [15] or indirectly by inhibition of a repressor. If the latter is the case we would expect to see expansion of A-box mediated repression into ventral lateral regions. In *Egfr* mutants, repression of the stripe was expanded ventrally, which we assayed by relating the reporter gene expression to the domain of *vnd* expression (*vnd* is expressed ventral to *ind*, in ventrolateral regions of the embryo) (Figure 6H). When the reporter was assayed in a wild-type background (i.e. *yw*), it extended about 8 cells above the dorsal border of *vnd* (Figure 6B). However, in *Egfr* mutants, strong expression of the stripe was only visible up to the ventral border of *vnd* (Figure 6H and I) and in some cases weak expression extends above the dorsal border of *vnd* (data not shown). These results suggested that the repressor binding the A-box element is itself inhibited by Egfr signaling. In the absence of Egfr signaling, repression is unrestrained and expands ventrally toward the ventral border of *vnd*.

Ajuria et al. [38] reported that the *ind* expression domain was slightly expanded in the absence of maternal *cic* transcript (*cic^1^/cic^1^*females). We introduced the *A-box.eve.stripe3/7.A-box* reporter into the *cic^1^/cic^1^* mutant background. Reporter expression was expanded into dorsal regions suggesting that repression activity was lost, however anterior-posterior patterning is severely compromised in *cic^1^/cic^1^* mutants (data not shown).

To examine whether Grh-mediated activation and Cic-mediated repression through the A-box might be linked in general, we examined other genes regulated by Cic to determine whether they might also be regulated by Grh. In Ajuria et al, they found that Cic binding sites which are similar to the A-box binding sites are found in several other CRMs and mediate Cic-dependent repression. We looked at one of these genes, *huckebein* (*hkb*), in *grh glc* mutant embryos to test the idea that Grh might act as a general activator for CRMs containing an A-box-like site (Figure 6E). We did not see an effect on *hkb* expression, suggesting that Grh activation via the A-box binding site does not act to regulate *hkb* expression (or other activators that support *hkb* expression). Our results suggest that Cic and Grh may work coordinately through the A-box but that they likely have independent binding sites/targets as well (see Discussion).

### Dpp signaling mediates repression that is independent of the repression mediated by the A-box elements

We found that Egfr signaling modulates A-box mediated repression, but we also investigated whether Dpp signaling functions through the A-box as previous evidence had shown a relationship between TGF-β signaling and *ind* expression [12], [17]. If the A-box repressor is a Dpp target gene or is regulated by one of the Dpp target genes we might expect to see a change in our repression activity upon modulation of TGF-β signaling. We introduced the *eve.stripe3/7* CRM flanked by the two A-box sites into *brk sog* double mutants, in order to assay the A-box repressor activity in a background with ectopic Dpp signaling. Brinker (Brk) and Sog both act to restrict Dpp signaling activity to the dorsal most regions of the embryo [39], [40]. The *brk* gene encodes a transcription factor that functions to repress transcription of Dpp target genes; in turn, the *sog* gene encodes an extracellular Dpp binding protein which acts both as a direct Dpp antagonist and is also required for high level Dpp signaling in the dorsal midline. In *brk sog* double mutants, ectopic Dpp is observed in lateral regions of the embryo and at the same time *ind* expression is also diminished [12] (Figure 6J). If the A-box repressor is a Dpp target gene or is regulated by one of the Dpp target genes, we would expect to see an expansion of the repression domain. However, we did not observe a significant change in the repression activity in this mutant background (Figure 6 K and L, compare with 6 B and C). This suggested that the A-box repressor acts independently of Dpp and its target genes. Dpp and its targets may still play a role in repression of *ind* via other unidentified binding sites.

**Figure 7:**
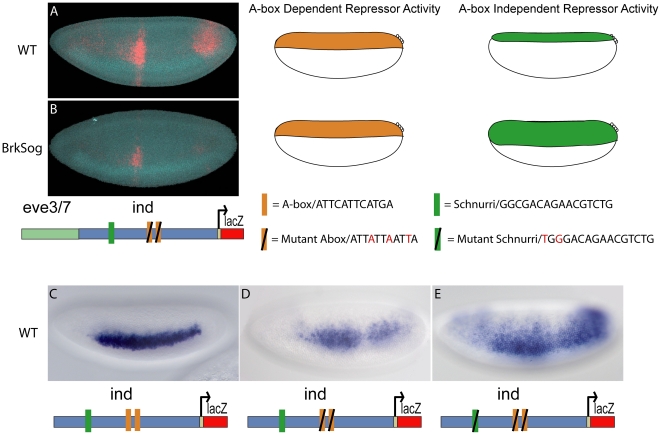
Dpp dependent repression is mediated via the Schnurri (SMM) binding site and is independent of A-box repression. A-box independent repression is observed in dorsal-most regions of the embryo in the *eve.stripe3/7-ind-mutant-abox* reporter construct. This construct was introduced into the *brk sog* mutant background and analyzed by in situ hybridization: *lacZ* (red) and *vnd* (blue) are shown in a WT embryo (A) and a *brk sog* mutant (B). The schematic at the bottom of the embryos shows the construct that was used to drive expression. The cartoon embryos on the right show where the designated repressors possibly expressed. (C) 1.4 kB *ind* CRM drives expression of a lateral stripe of 5–7 cells in width comparable to *ind* expression. (D) 1.4 kB *mut-A-box-ind* CRM drives expression of 7–10 cell width lateral stripe that is diffuse, weak, and expanded compared to the *ind* CRM. (E) 1.4 kb *mut-A-box-mut-SMM-ind* CRM drives expression of 12–15 cell width lateral stripe that is expanded compated to the *ind* CRM and *mut-A-box-ind* CRM.

When we analyzed expression supported by the *eve.stripe3/7-ind-mutant-A-box* reporter construct, we noted repression in the dorsal-most part of the embryo despite the lack of A-box sites (Figure 1D and 6M). To investigate whether this particular repression activity was dependent on Dpp signaling, we assayed this reporter in *brk sog* double mutants. If this repression in dorsal-most regions of the embryo is dependent on Dpp signaling, we would expect to see an expansion of the repression into dorsal-lateral regions of the embryo. This was what we observed: the repression supported in *brk sog* mutants was present in a more broad domain, expanded dorsally well beyond its limit in wild-type embryos (Figure 7B). These results suggested that this repression in dorsal-most regions is dependent on *Dpp* signaling and is independent of the repression mediated by the A-box elements.

Schnurri is a Dpp target gene that is expressed in dorsal regions of the embryo [41], [42], [43]. It binds to DNA via the Mad and Medea binding sites forming a Schunurri/Mad/Medea (SMM) protein complex that mediates repression [24], [25]. A SMM binding site is located in the *ind* CRM; it is possible that Dpp signaling mediates repression of *ind* via this binding site. In order to test this hypothesis we mutated the SMM site (Mad binding component) in an *ind* CRM that contained two mutant A-box sites and found that the expression pattern is further expanded (Fig. 7 compare D and E).

Thus, our results suggest that two distinct dorsally-localized repression activities refine *ind*, one dependent on Dpp signaling and the other independent of this signaling. This view is supported by the fact that ectopic Dpp is able to repress *ind* and yet loss of Dpp has no affect on its expression [12]; we suggest that A-box mediated dorsal repression can compensate in the absence of Dpp. When Dpp signaling is overexpressed in a permissive environment that supports activation of its target genes, its presence is sufficient to repress *ind* in a Dpp-dependent fashion [17], but when Dpp signaling is lost, repression through a Dpp-independent mechanism (i.e. A-box repressor) is still able to restrict *ind* thus an expanded pattern is not observed.

## Discussion

**Figure 8:**
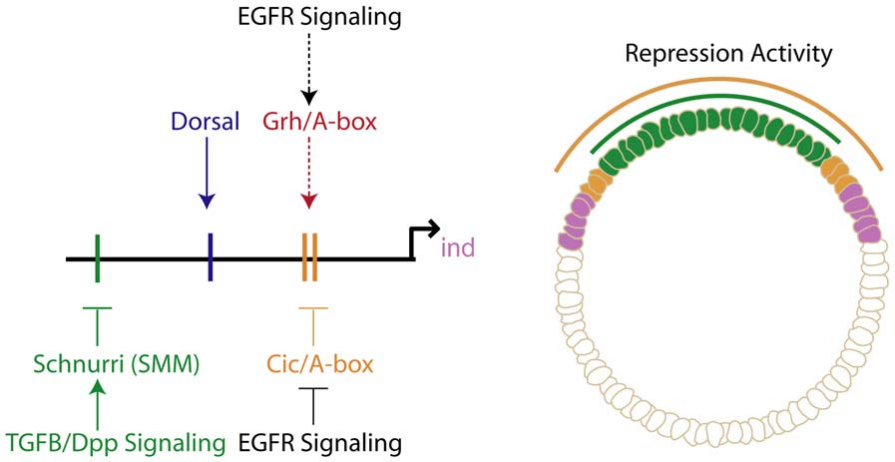
Model for transcriptional regulation of *ind* expression. Our model is based on a compilation of this study and other studies suggesting that several transcription factors and signaling pathways interact to specify the *ind* pattern. This is only a partial model and does not include all the factors that delineate the ventral borders of *ind*. The dorsal border is established by two tiers of repression: one mediated by the A-box binding site/Cic and the other mediated by a Dpp dependent repressor/Schnurri (SMM). Activation is mediated by Grh via the A-box binding site and by Dorsal via Dorsal binding sites. The depiction shows the repressor activity relative to *ind* expression. Schnurri repression activity is limited to dorsal-most regions of the embryo. The A-box/Cic activity is found in dorsal and dorsal–lateral regions. The dashed lines indicate interactions that are still unclear.

We analyzed the A-box sequence and showed it is both necessary and sufficient for repression of *ind* in dorsal-lateral regions and sufficient for dorsal-most repression. Through DNA affinity chromatography and mass spectrometry, we identified several binding factors many of which are involved in chromatin remodeling. One of the factors we identified, encoded by the *grh* gene, was previously shown to act as an activator as well as a repressor throughout development and during wound response [29], [44]. We showed Grh protein binds the A-box binding site in vitro. Since mutagenesis of the A-box sites within the *ind* CRM leads to decreased reporter expression and *ind* endogenous expression is also diminished in *grh* mutants, this data suggested that Grh drives activation of *ind* through the A-box; we note however that we cannot dismiss an additional role for Grh through other sequences in the *ind* CRM. We also demonstrated the repressive function of the A-box is restricted by Egfr signaling and is independent of Dpp signaling. In turn, we found, repression mediated by Dpp signaling does impact *ind* in dorsal-most regions of the embryo and possibly acts through the SMM binding site, not the A-box. Collectively, our results show interactions between several signaling pathways and transcription factors are necessary to establish the *ind* expression pattern (Figure 8).

### Combinatorial action of Grh and Dorsal likely support *ind* activation

Other studies have shown combinatorial interactions are necessary to support patterns of gene expression along the DV axis. For instance, one study showed Dorsal and Zelda function together to produce the broad lateral domain of *sog*. Mutation of either the Dorsal sites or the Zelda sites in the *sog* CRM produced a pattern that was narrower than the wild-type expression pattern. It was concluded that both Dorsal and Zelda must be present to produce a proper Sog pattern [9]. It is also well appreciated that Dorsal can act cooperatively with the bHLH transcription factor Twist to support expression in ventral and ventrolateral regions of the embryo [8]. We propose Grh and Dorsal act together to support the *ind* expression pattern. While the *ind* CRM containing a mutant Dorsal site did support some expression, the expression pattern contained a gap and was weaker in posterior regions; in contrast, in Dorsal mutants, *ind* expression is completely absent. This result may be explained if both indirect as well as direct functions for Dorsal are required to support *ind* expression. For instance, Dorsal has other target genes including *rho*, which is required to support Egfr signaling [45], [46]. Furthermore, mutation of the A-box/Grh binding site within the *ind* CRM caused expression of the reporter that was expanded dorsally and weak, suggesting this site mediates repression and also activation. Similar to Dorsal mutants, the phenotype we observed when we mutated the A-box sites is different than the phenotype in the Grh mutants, thus we cannot rule out that Grh may act through other sites as well as the A-box and/or that Grh may act indirectly to influence *ind* expression by regulating the expression of other transcription factors. We propose a model most consistent with the current data which is that *ind* is activated in regions where Dorsal is present as well as optimal levels of Grh (see below); it is then refined by Snail and Vnd in ventral regions and Cic and Schnurri/Mad/Medea (SMM) in dorsal regions (Figure 8).

### Egfr signaling may act to regulate the activity of both Cic repressor as well as Grh activator to support *ind* expression

*grh* and *cic* genes are both maternal and ubiquitously expressed, thus, another input is necessary to explain how localized expression of *ind* is supported. This positional information could be provided in part by competition between Grh and Cic proteins for the A-box binding site and in part by ventrolaterally-localized Egfr signaling. A model in which Egfr signaling supports activation of *ind* via inhibition of a ubiquitous repressor (e.g. Cic) is supported by our results which demonstrate that A-box mediated repression is expanded in *Egfr* mutants. A recent study also showed expanded expression of an *ind* CRM fragment reporter in *ras cic* double mutants in which neither Egfr signaling or Cic repressor is present, suggesting that Egfr may function by inhibition of an “inhibitor” to promote activation [38]. This data suggests that the putative A-box repressor, Cic, may not be dorsally localized but that its activity is regulated by Egfr signaling which provides the positional information necessary for a sharp boundary. However, the domain of dpERK activation (as detected by anti-dpERK, an antibody to the dual-phosphorylated from of ERK) does not exactly overlap with the *ind* expression domain at cellularization (data not shown), as would be expected in the simplest model.

Ajuria et al. suggested that Egfr signaling supports *ind* expression through inhibition of Cic, and we add that it is also plausible Egfr signaling impacts activation of *ind* through Grh. In fact, a recent study showed that Grh activity during wound response is modulated by ERK signaling [44]. Specifically, they found both unphosphorylated and phosphorylated Grh can bind DNA and act as an activator. The former is used during normal development of the epidermal barrier and the latter is used to overcome a semi-dormant state during wound response. Another study showed the tyrosine kinase Stitcher activates Grh during epidermal wound healing [47]. In the early embryo Grh may be phosphorylated by Egfr signaling to support activation of *ind* through the A-box binding site. We suggest that phosphorylation of both Grh as well as Cic by Egfr signaling can act as a switch to help fine-tune the expression of *ind*.

### Grh and Cic function coordinately through the A-box but likely also have independent actions at other distinct binding sites

We investigated whether a relationship between Grh activation and Cic repression was used in regulation of other genes containing A-box or Cic binding sites. We found that one other Cic target gene, *hkb*, was unaffected in Grh mutants. As the A-box site (WTTCATTCATRA) is larger than the Cic consensus binding sequence [T(G/C)AATGAA, complement TTCATT(G/C)A] defined by Ajuria et al, it is possible that Grh needs the full A-box site to bind. The full A-box sequence is not present in the *hkb* CRM, but Cic binding may be facilitated by a partial sequence (i.e., TGAATGAA). Alternatively, it is possible that a role for Grh and/or Cic at the A-box is context dependent. For instance, Grh-mediated activation may be a necessary input to support *ind* expression but not for the support of *hkb*, which also receives activation input from Bicoid and Hunchback transcriptional activators and is expressed in the pre-cellularized embryo.

Other studies have suggested that Grh acts to repress transcription of *fushi tarazu (ftz)*, *dpp*, and *tll* in the *Drosophila* embryo [29], [33], [48], but our study is the first to identify a role for Grh-mediated gene activation in the early embryo, in support of dorsoventral patterning. Previous studies had shown that Grh can function as an activator at later embryonic stages [48], [49]. One analysis identified Grh (also called NTF-1 or Efl-1) biochemically using an element from the *dpp* early embryonic CRM, however the *dpp* expression domain was unchanged in the *grh* mutants [29].

Another recent study also showed Grh binds to sites that are similar to Zelda binding sites [34]. Zelda and Grh each showed stronger affinity for different variations of the shared consensus sequence, but in vitro studies showed they also competed for binding. Harrison et al. proposed that as levels of Zelda increase it is able to compete against Grh for binding sites and cause activation of the first zygotic genes. Competition at the same binding sites results in a cascading effect in which ubiquitous activators regulate genes in a temporally related manner. They proposed Grh functions first to silence gene expression; while, alternatively, our data is more consistent with a model in which Grh mediated activation follows that of Zelda. *ind* is considered a “late” response gene as it appears at mid stage 5 (nc 14), at the onset of cellularization, whereas Zelda was shown to support gene expression earlier at nc 10 [10].

It is possible that Grh competes for binding to a variety of sites (not only those recognized by Zelda), and that this competition influences gene activation/repression. At the A-box sequence, Cic and Grh may compete to help establish a sharp boundary; unfortunately, the Cic binding to the A-box sequence demonstrated previously *in vitro* was quite weak [38], so this competition is best examined *in vivo* in future studies.

### Tiers of repression are likely a common mechanism to ensure robust patterning

This study found there is yet another tier of repression activity that is independent of the A-box mediated repression. Analysis of the *eve.stripe3/7-ind-mutant-A-box* reporter construct revealed that, while dorsal-lateral repression was lost, there was still repression in the dorsal-most part of the embryo. This led us to reason that other binding sites in the *ind* CRM, independent of the A-box binding site, mediate repression. Previous research showed ectopic TGF-β/Dpp signaling can repress *ind* expression, and therefore we hypothesized the repression activity we observed in dorsal-most regions of the embryo may be regulated by Dpp signaling.

Our results suggested that the Dpp dependent repression supports repression in the dorsal most part of the embryo and not in dorsal lateral regions of the embryo. We would not expect to see an expansion of the *ind* domain in the mutants affecting only this dorsal-most repressor, thus we mutated the SMM site in the context of two mutant A-boxes and found that the expression pattern was expanded into dorsal regions of the embryo. However, when we mutated the A-box sites, we observed expansion of *ind* more dorsally into dorsal-lateral regions but expression was absent in dorsal-most regions. It is possible the embryo can tolerate a slight expansion of *ind* into dorsal lateral regions of the embryo but expansion of *ind* into the non-neurogenic ectoderm is detrimental. Thus, two tiers of repression have developed to insure that expression of *ind* is limited to the neurogenic ectoderm. We suggest that partially redundant repressor mechanisms are more common than appreciated, because in contrast to activation it is difficult to track repression activity.

### Chromatin factors may play a role in regulating *ind* via the A-box

Epigenetic changes to DNA and chromatin remodeling have been shown to be vital in repression and activation of genes that define structures in late stages of *Drosophila* development. For example, Polycomb group genes silence the homeotic genes of the Bithorax complex, which control differentiation of the abdominal segments [50]. To date, little is known regarding how/if chromatin factors play a role in early development of *Drosophila* embryos. Here we presented evidence that several chromatin-related factors bound an A-box affinity column but did not bind a column containing the mutant A-box element (Figure S2). Although several of these factors did not bind to the A-box element alone when tested by EMSA, it is possible that they bind indirectly via a larger complex. One of these factors Psq has been implicated in both silencing and activation via the Polycomb/Trithorax response elements [51], [52]. Independently, Psq was recently found to positively regulate the Torso/RTK signaling pathway in the germline, while being epistatic to *cic* a negative regulator of the Torso signaling [53]. It is possible that some of these factors play a role in regulating *ind* via the A-box element, which would suggest a role for chromatin remodeling early in development - an avenue which is worth pursuing in future studies.

## Materials and Methods

### Fly stocks and mutant analysis

*Drosophila melanogaster* flies of the background *yw* were used as wild-type. Transgenic reporters were created by P-element-mediated transformation using standard methods (A-box.*eve.stripe3/7*.A-box) and site-directed transformation into the 86FB strain (all other transgenic lines) *FRT 42D grh^IM^* and *FRT 42D GFP* fly stocks were used for creating germline clones [54]. The *grh^B37^* allele was also used [49] and recombined with *FRT 42D* in order to facilitate generation of germline clones. *Df(2R)Pcl7B/Cyoftzlacz* is a deficiency mutant that removes the *grh* locus, and was used to eliminate the possibility that a second-site mutation within the *grh^IM^* background was responsible for loss of *ind*. *FRT 42D grh^IM^/Cyoftzlacz; A-box-eve.stripe3/7-A-box* flies were used in the A-box repression assay (Figures 6F and H, respectively). The CyO ftzlacZ marked balancer was used to distinguish zygotic genotype in crosses; however we found that the frequency of ftzlacZ+ embryos was very low in the embryos devoid of maternal *grh* therefore assay of zygotic genotype was inconclusive. It is possible that *grh* may be required to support *ftz* expression (M.G. and A.S., unpub. obs.), and other studies have identified a later role for *grh* in supporting *ftz* expression [48]. The zygotic genotype may relate to the variability observed in the *ind* expression phenotype.

*Toll^rm9^/TM3Ser* and *Toll^rm10^/TM3Sb* fly stocks were used to generate transheterozygous *Toll^RM9^/Toll^RM10^* females, and *pipe386/TM3Sb* and *pipe664/TM3Sb* fly stocks were used generate transheterozygous *pipe386/pipe664* females, as previously decribed [2]. Homozygous *cic1/cic1* females were obtained from a *cic1/TM3SbSer* stock [55]. Virgin females were obtained from each of these crosssed and mated to males containing the A-box repression reporter (*A-box-eve.stripe3/7-A-box-ep-lacZ*) (this work, see below). *brk^M68^sog^Y506^* [40], [49] mutants were used to create *brk^M68^sog^YS06^/FM7ftzlacZ*; *A-box-eve.stripe3/7-A-box* and *Egfr^f2^* [56] mutants were used to create *Egfr^f2^/CyoftzlacZ; A-box-eve.stripe3/7-A-box* fly stocks, which were used in the A-box repression assay (Fig. 6).

### Plasmid construction

The *A-box*-*eve.stripe3/7-A-box* reporter was created by PCR using the following primers MG 1 (5′-gtgcggccgcAGCGC**ATTCATTCATGA**GGCCAggacacaaggatcctcgaaatcgaga-3′) and MG 2 (5′-gtgcggccgcACACT**TCAGAATGAAT**ACATCgaaggaacgagctcgtaaaaacgtgaa-3′) and was cloned into pCasper using the Not I site. The chimeric CRM were created by cloning the modified *ind* CRM into a pGemT-easy vector containing the *eve.stripe3/7* CRM using the Spe1 site. The *eve.stripe3/7* CRM [26] was PCR amplified using MG 48 (ggacacaaggatcctcgaaat) and MG 49 (gaaggaacgagctcgtaaa). A fragment containing both CRMs in tandem was subsequently cloned into the pLacZattB vector using the Not 1 site.

The mutant CRMs were created by PCR site directed mutagenesis using the following primers: A-box1: MG 87 (caggcagtgcagcgcattattaattaggccaattc) and MG 88 (gaattggcctaattaa-ttaatgcgctgcactgcctg); A-box2: MG 99 (ctgaagaggttctgcacttcaggatgtattaattaattaagtgtcttccacgcg) MG 100 cgcgtggaagacacttaattaattaatacatcctgaagtgcagaacctcttcag); Dorsal: MG 106 (caggccca-aagaacctgacccaatttcccagccttgatg) and MG 107 (gtccgggtttcttggactgggttaaagggtcggaactac). SMM: MG 234 (ggacttatatgcccttgggacagaacgtctggac) and MG 235 (gtccagacgttctgtcccaagggcatataagtcc).

### *In situ* hybridization

Embryos were collected, fixed, and subsequently hybridized with dioxygenein-UTP, biotin-UTP or fluorescein-UTP labeled antisense probes as previously described [57], [58]. Probes were made by PCR from genomic DNA extracted from *yw* male flies. Images were collected using bright field or confocal microscopy.

### Preparation of nuclear extracts

Nuclear extracts were prepared using 45 grams of 0–6 hour embryos using a modified version of the protocol described in [59]. Frozen embryos were ground in liquid nitrogen using a mortar and pestle. The ground embryos were resuspended in 200 ml of buffer containing 25 mM Hepes pH 7.6, 10 mM KCl, 1.5 mM MgCl_2_, 0.1 mM EDTA, and 1× Roche proteinase inhibitor. The solution was homogenized using a dounce homoginizer, and subsequently was centrifuged at 10,000 g for 10 minutes. The supernatant was removed and the pellet was resuspended in 150 ml of buffer containing 25 mM Hepes 7.6, 10 mM KCl, 1.5 mM MgCl_2_, 0.1 mM EDTA, 25% glycerol and 1× Roche Proteinase inhibitor. 15 ml of 5 M NaCl was added. The solution was mixed for 20 minutes at 4°C. The solution was centrifuged at 15,000 g for 20 minutes. The resulting supernatant was the nuclear extract.

### Affinity chromatography and mass spectrometry

Dialyzed extracts were partially purified by eluting off a heparin column using 0.3 M–1.5 M KCl. Fractions from the heparin column were assayed for A-box binding activity using EMSA. The fractions with activity (i.e., 0.9 M–1.1 M KCl) were combined and dialyzed. Half of the sample was run on an A-box affinity column (gatctgt**attcattcatga**agtgtcttc) and half was run on a mutant A-box affinity column (gatcgcagcgc**attaattaatta**ggc). Columns were prepared and run according to previously described protocols [60]. The fractions were tested for activity using EMSA and binding proteins were identified using GelC/mass spectrometry. Standard in gel trypsin digest with reduction and alkylation was used to process samples for mass spectrometry. A Thermo Finnigan Orbitrap was used for mass spectrometry of samples. The Scaffold program was used to identify targets. Positives were differentiated from false positives by comparing the A-box column list to the mutant A-box column list. The list was also cross-referenced to a list of all previous characterized transcription factor or factors containing a predicted DNA-binding domain. The list of putative transcription factor was obtained from FlyTF.org [61].

### Elecrophoretic Mobility Shift Assay

The following oligos were used for the EMSA: A-box (gatctgtattcattcatgaagtgtcttc) and mutant A-box (gatctgtattaattaattaagtgtcttc), and standard labeling methods with γ^32^P-ATP were used. The following buffer and conditions were used for tracking the activity during affinity chromatography: 10 mM Tris pH 7.5, 5% glycerol, 15 M sucrose, 2 mM EDTA, 50 mM DTT, 200 mM KCl, 1% nonidet P-40, 5 ug/ul BSA, 0.3 ug/ul polydIdC 1× Roche complete protease inhibitor, 100 fmol of labeled oligo, and 1 ul of extract in a 25 ul reaction. For the testing of candidate genes 25 mM Hepes pH7.9, 100 mM KCL, 1 mM DTT, 1% polyvinyl alcohol, 1% nonidet P-40, 0.1% BSA, 10% glycerol, 0.25 uM calf-thymus DNA, 50 fmol of labeled oligo and 1 ul of reticulocyte in vitro translated protein was added to a reaction of 15 ul total volume. Proteins were prepared using the TNT T7 Quick Coupled Transcription/Translation System from Invitrogen. The reactions were incubated for 30 minutes on ice and then resolved on either 6% or 4% native polyacrylamide gels containing 0.5× TBE.

## Supporting Information

Figure S1 **Flow-chart outlining the protocol used to purify factors that bind the A-box element.** First we created nuclear extracts from 0–6 hour embryos. Then we fractionated the sample using a heparin column and tested the fractions for specific A-box binding. We affinity purified the fractions that contained specific A-box activity using an A-box column and a mutant A-box column. We again tested for A-box binding and identified factors bound to both columns using mass spectrometry.
(TIF)

Figure S2 **Overabundance of Chromatin remodeling and histone modifying factors found binding to A-box column versus the mutant A-box column.** The percentage was calculated by dividing the number of factors in each specific category by the total number of factors found to bind only the A-box column or mutant A-box column. The number on the bar corresponds to the number of factors in each specified category.
(TIF)

Figure S3 **EMSA shows binding of Grh to the A-box binding site.** Rabbit reticulolysates were used to in-vitro translate the Grh protein and EMSA was preformed using γ^32^P-labeled A-box oligonucleotides. Non-specific binding indicated by the black arrows on the left was detected in the lysate alone. This binding was diffuse throughout the column. The Grh binding was strong and sharp (indicated by the black arrow on the right), and was only seen when the A-box oligonuleotide was used and not the mutant A-box oligonucleotide.
(TIF)
