# Supplementary figures and images for: Lateral Gene Expression in Drosophila Early Embryos Is Supported by Grainyhead-Mediated Activation and Tiers of Dorsally-Localized Repression
Source: PLoS One. 2011 Dec 22;6(12):e29172. doi: 10.1371/journal.pone.0029172 (PMC3245246; doi:10.1371/journal.pone.0029172)

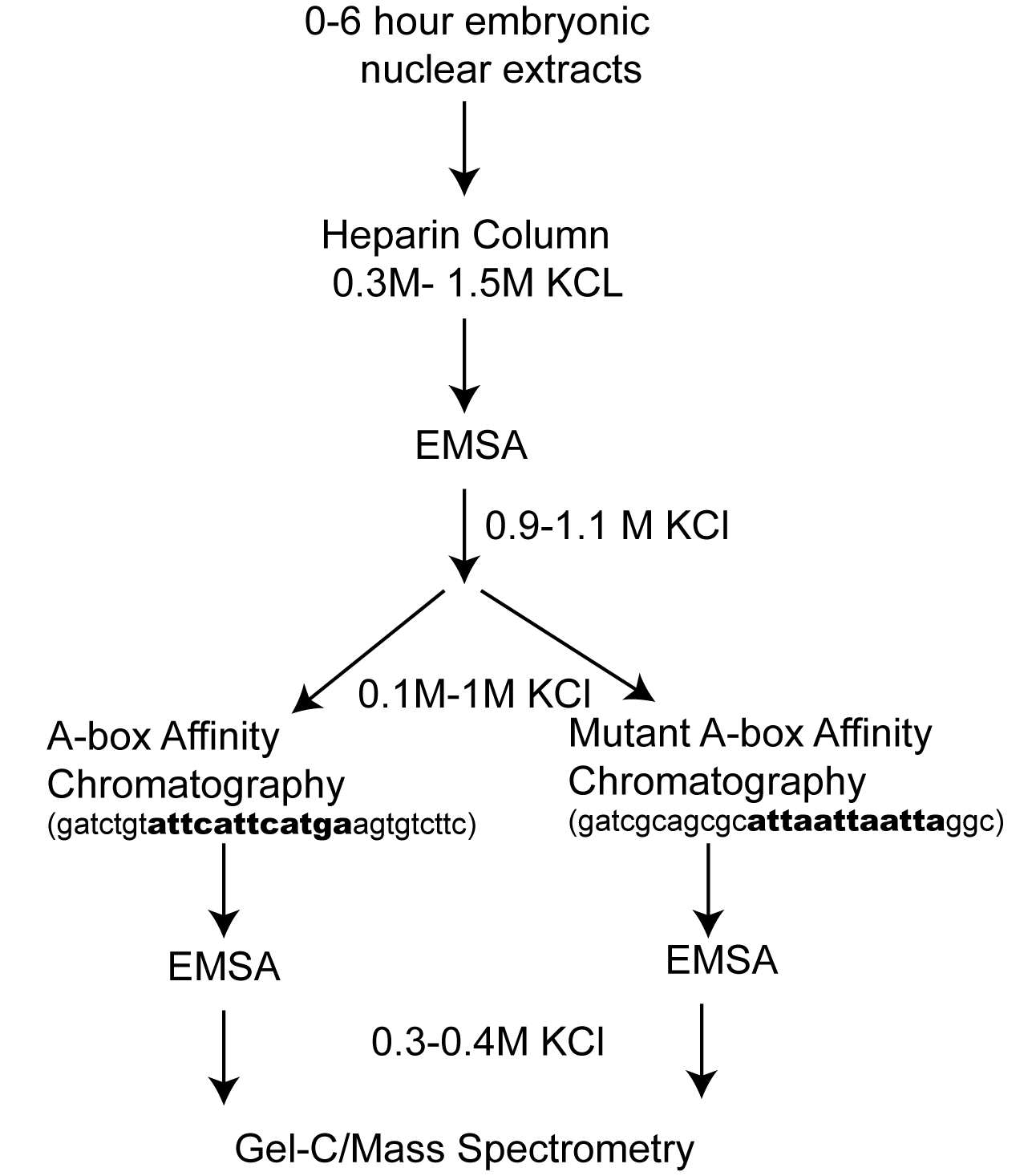

Supplement: Figure S1 — Flow-chart outlining the protocol used to purify factors that bind the A-box element. First we created nuclear extracts from 0–6 hour embryos. Then we fractionated the sample using a heparin column and tested the fractions for specific A-box binding. We affinity purified the fractions that contained specific A-box activity using an A-box column and a mutant A-box column. We again tested for A-box binding and identified factors bound to both columns using mass spectrometry. (TIF) [file pone.0029172.s001.tif]

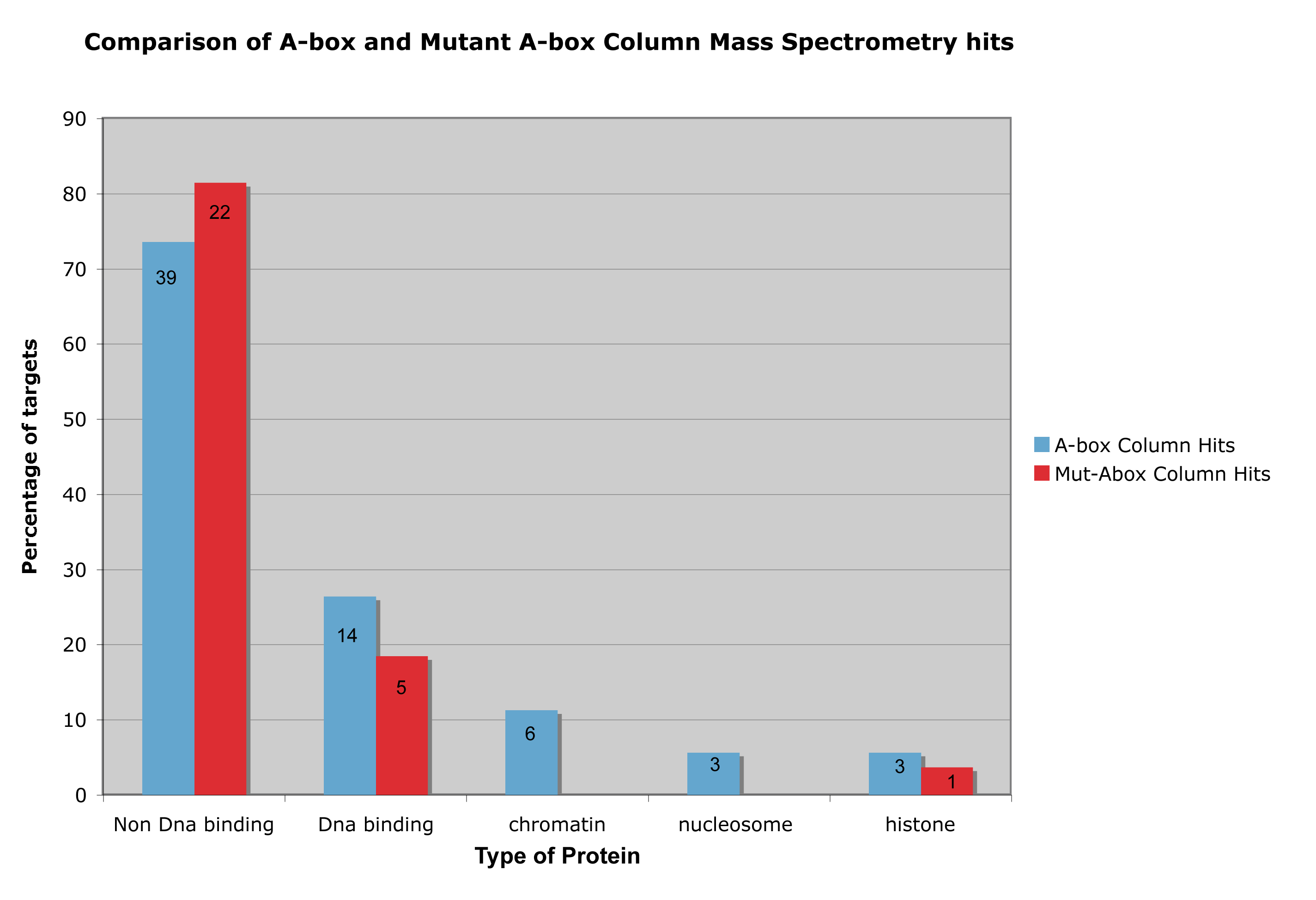

Supplement: Figure S2 — Overabundance of Chromatin remodeling and histone modifying factors found binding to A-box column versus the mutant A-box column. The percentage was calculated by dividing the number of factors in each specific category by the total number of factors found to bind only the A-box column or mutant A-box column. The number on the bar corresponds to the number of factors in each specified category. (TIF) [file pone.0029172.s002.tif]

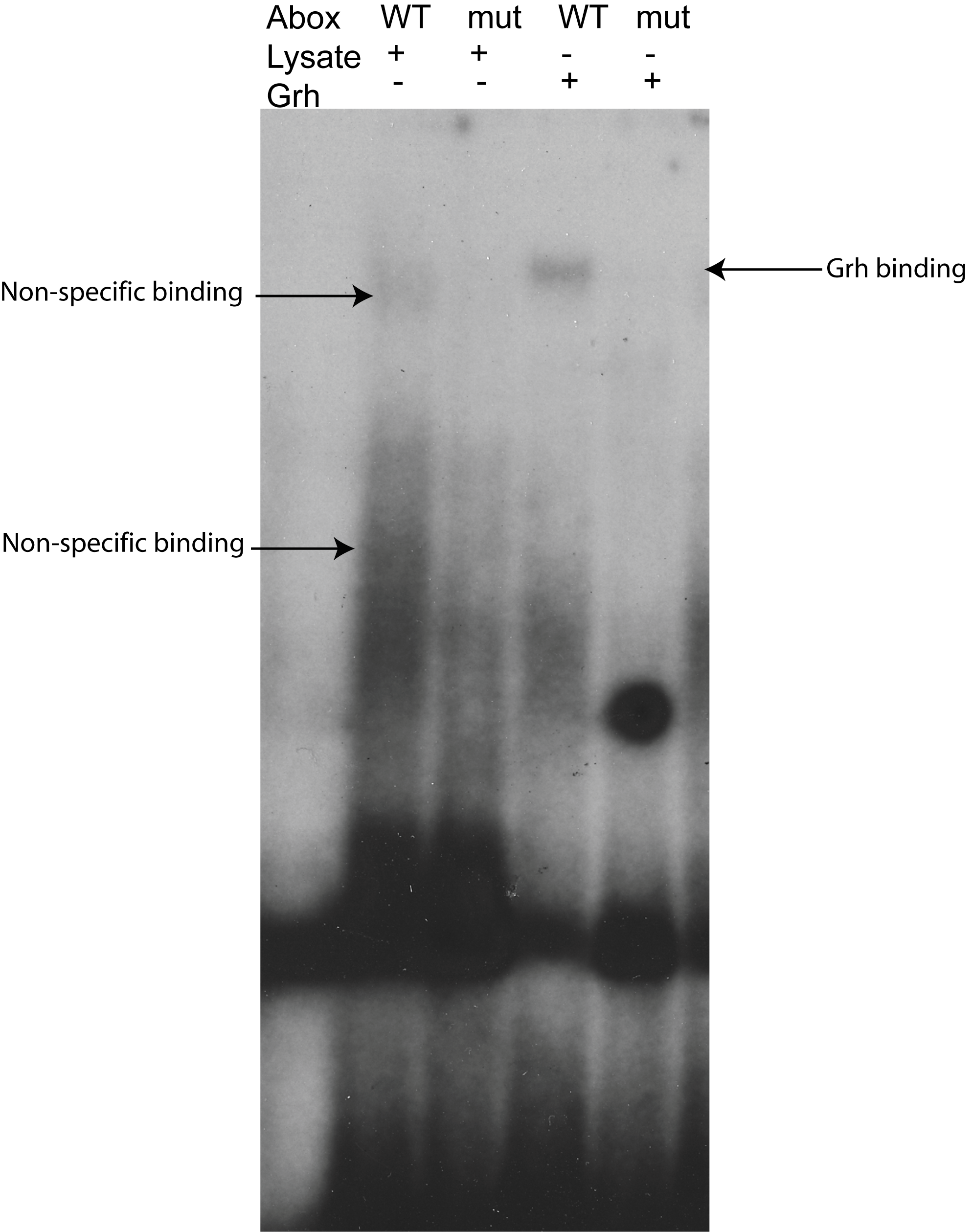

Supplement: Figure S3 — EMSA shows binding of Grh to the A-box binding site. Rabbit reticulolysates were used to in-vitro translate the Grh protein and EMSA was preformed using γ32P-labeled A-box oligonucleotides. Non-specific binding indicated by the black arrows on the left was detected in the lysate alone. This binding was diffuse throughout the column. The Grh binding was strong and sharp (indicated by the black arrow on the right), and was only seen when the A-box oligonuleotide was used and not the mutant A-box oligonucleotide. (TIF) [file pone.0029172.s003.tif]
